# Sustainable Composites: Analysis of Filler–Rubber Interaction in Natural Rubber–Styrene–Butadiene Rubber/Polyurethane Composites Using the Lorenz–Park Method and Scanning Electron Microscopy

**DOI:** 10.3390/polym16040471

**Published:** 2024-02-08

**Authors:** Arthur Pimentel De Carvalho, Harison Franca Dos Santos, Gabriel Deltrejo Ribeiro, Carlos Toshiyuki Hiranobe, Danielle Goveia, Elmer Mateus Gennaro, Leonardo Lataro Paim, Renivaldo José Dos Santos

**Affiliations:** 1 Department of Engineering, Faculty of Engineering and Science, Sao Paulo State University (UNESP), Rosana 19274-000, SP, Brazil; arthur.pimentel@unesp.br (A.P.D.C.); harison.franca@unesp.br (H.F.D.S.); gabriel.deltrejo@unesp.br (G.D.R.); carlos.hiranobe@unesp.br (C.T.H.); leonardo.paim@unesp.br (L.L.P.); 2 Department of Science and Technology, Institute of Sciences and Engineering, Sao Paulo State University (UNESP), Itapeva 18409-010, SP, Brazil; danielle.goveia@unesp.br; 3 Department of Aeronautical Engineering, Engineering School, Sao Paulo State University (UNESP), São João da Boa Vista 13876-750, SP, Brazil; elmer.gennaro@unesp.br

**Keywords:** composites, filler–rubber interaction, Lorenz–Park, polyurethane waste, shoe sole

## Abstract

This study examined micronized polyurethane residues as a reinforcing filler in elastomeric composites made from natural rubber (NR) and styrene–butadiene rubber (SBR). Due to growing environmental concerns, this research aimed to find sustainable alternatives to synthetic materials. The results indicated that adding micronized polyurethane improved the mechanical properties of the composites, reinforcing the polymer matrix and increasing the cross-link density as a barrier against solvents. The composites met the requirements for industrial applications, though; at 40 phr of polyurethane filler, material deformation was reduced, indicating saturation. FTIR analysis confirmed the homogeneity of the materials without chemical reactions, while electron microscopy revealed an increase in the number of particles and irregularities with the filler. The composite with 10 phr showed a lower volume loss in abrasion resistance, meeting the standards for soles. The composite with 30 phr of polyurethane achieved the best results without the filler’s saturation and met the footwear industry’s requirements. The results show the potential for sustainable practices in industry using this elastomeric blend.

## 1. Introduction

Growing awareness of the environmental issues resulting from the excessive utilization of synthetic materials has focused the world’s attention on sustainable materials, along with a circular economy strategy that encompasses recycling pathways [1]. The need for new materials has led to the search for raw materials that can be reused and have sustainable characteristics without causing negative impacts on the environment. This scenario requires the development of new alternative materials to replace those manufactured conventionally [2]. Composite materials can represent an appropriate alternative to mitigate the adverse impacts of many materials at the end of their useful life. Natural or synthetic rubber raw materials, in which a material dispersed in its polymer matrix, such as polyurethane waste, can result in a sustainable composite.

The global polyurethane market was valued at USD 80 billion in 2022 and is projected to reach around USD 118.99 billion by 2032, with a compound annual growth rate (CAGR) of 4.10% over the forecast period from 2023 to 2032 [3]. The growing demand for this type of material is driven by the unique characteristics of polyurethane, which can be shaped into unconventional forms without sacrificing quality. Furthermore, polyurethane improves industrial and consumer goods, contributing to their comfort and usefulness, hence increasing their worth. The production of polyurethane involves carefully controlled chemical processes using polyols, diisocyanate, or polymeric isocyanate, as well as the intentional inclusion of additives and catalysts. These diverse compositions cater to the specific requirements of numerous industries, generating tailor-made industrial solutions using a broad spectrum of polyols [4]. Polyurethane products have applications in a wide variety of everyday consumer goods. For instance, polyurethane is a protective layer in stiff foam applications for walls and roofs and in flexible foam applications for furniture upholstery. Additionally, it is utilized as thermoplastic polyurethane (TPU) in medical equipment and footwear.

In addition, polyurethane is employed in applying coatings, sealants, and adhesives, as well as in the flooring and interiors of motor vehicles. Polyurethane is in high demand in the refrigeration and construction industries due to its outstanding performance as a thermal insulator [5]. The extensive use of polyurethane across various applications has significantly increased consumption, causing detrimental effects on the environmental balance. This is primarily attributed to the prolonged longevity of these objects, which are commonly disposed of in landfills or incinerated in numerous countries [6].

Various studies have been carried out to investigate a composite material capable of using polyurethane as a filler while maintaining the integrity of its mechanical, thermal, and electrical properties. Sułkowski et al. [7] examined the behavior of compounds derived from polyurethane and rubber waste from tires under varying temperature conditions. This analysis can provide essential insights into the thermal stability of compounds, which is crucial for understanding their application potential, particularly in the automotive industries. In the same research segment, Hu et al. [8] developed a new type of elastomer with polyurethane that can be used in the next generation’s high-performance tires. This self-repairing, recyclable, and heat-resistant material delivers a comprehensive performance equivalent to existing eco-friendly tires. In another segment of research, Gómez-Rojo et al. [9] analyzed the chemical, microstructural, and physical properties of polyurethane foam waste from different types of industries, intending to evaluate its potential use in construction materials. Furthermore, they suggested incorporating the waste into plaster, mortar, or concrete matrices to improve their thermal and acoustic insulation properties.

In addition to the mentioned research, Cachaço et al. [10] analyze the potential use of recycled rubber from unusable tires to create composite polyurethane foam materials with promising properties for various applications. The authors explore the development of two specific products: floating trays for cleaning polluted water and compression-absorbing buoys to cushion shocks between ships and docks. They demonstrate that polyurethane formulations and the rubber content can be modified to achieve the desired characteristics for each application. Balan et al. [11] investigated the effect of silane coupling agents on the mechanical and thermal properties of thermoplastic polyurethane (TPU)/natural rubber (NR) blend composites reinforced with coconut shell powder (CSP). This study showed that silane coupling agents could effectively enhance the mechanical and thermal properties of TPU/NR blend composites reinforced with CSP. Furthermore, Zhang et al. [12] demonstrated the feasibility of using a composite modifier of rubber-polyurethane powder to improve the performance of cold patch asphalt, offering a promising approach to enhance the durability and longevity of pavement repairs. Thus, it can be noted that polyurethane can be employed as a filler and a matrix.

This study investigated various methods to enhance the mechanical characteristics of NR/SBR composites. This study also examined the potential of utilizing micronized polyurethane residues to enhance the strength of elastomeric matrices. The generally acknowledged Lorenz–Park equation was utilized due to its firmly established capacity to ascertain the interfacial interaction between the matrix and filler in composites.

## 2. Experimental Process

### 2.1. Materials

Brazilian light crepe natural rubber (NR) and synthetic rubber of the styrene-butadiene type (SBR 1502) were supplied commercially in Poloni, SP, Brazil. The polyurethane (PU) residue was purchased from the company Metalfrio in Três Lagoas, MS, Brazil. According to the manufacturer, its composition includes 165 parts of MDI-type isocyanate (2,2-diphenylmethane diisocyanate), 100 parts of polyether, and 14 parts of pentane gas as an expanding agent. Subsequently, this residue was ground and sieved until a particle size greater than 30 mesh. Vulcanization reagents like sulfur, benzothiazole disulfide (MBTS), zinc oxide, and tetramethylthiuram disulfide (TMTD) accelerators were bought from stores.

### 2.2. Composite Preparation

For making the composites, 50 phr NR and 50 phr SBR rubbers were set, along with the reagents to be used and the amount of PU residue (0, 10, 20, 30, and 40 phr) to be incorporated into the rubber mixture based on established parameters. Furthermore, tests were carried out with 50 phr of polyurethane residue. However, mixing the filler into the polymer matrix was difficult, which impacted the mechanical properties. The composites were prepared in an open two-roll mill from Makintec, model 379 m, at 65 °C and a friction ratio of 1:1.25, and their masses were measured in phr (per hundred rubber), according to ASTM D3182-21a [13]. The formulations used in this study are shown in Table 1.

Following the homogenization process, the mixture was allowed to settle undisturbed for 24 h, maintaining a consistent room temperature (25 °C). Afterward, the dough was reintroduced into the mixer to incorporate vulcanization accelerators and the cross-linking agent. After thoroughly mixing the components, the dough was left untouched for 4 h at room temperature. A small part of the sample was submitted for rheometric testing to determine the parameters. In contrast, the other portion of the sample was submitted to thermoforming by hydraulic press at 160 °C.

### 2.3. Rheometric Properties

The rheometric parameters were determined following the guidelines of ASTM D2084-19a, using an oscillatory arc of 1° and isotherms at 160 °C [14].

### 2.4. Density

The density of the composites was determined following ASTM D297-21 [15] and calculated by Equation (1):

(1) $$\rho =\frac{\rho_{L}\times m_{A}}{m_{A\;}{-m}_{B}}\;\;$$

where *ρ* is the sample density (g cm^−3^); *ρ_L_* represents the ethanol density (g cm^−3^); $m_{A}$ and $m_{B}$ are the mass (g) of the wireless sample in the air and the liquid, respectively.

### 2.5. Cross-Linking Density

To ascertain the cross-linking densities of the composites, firstly, 0.25 ± 0.05 g was submerged for five days in toluene. Upon measuring the masses of the dry sample, the sample swollen with solvent, and the sample after swelling, the recorded values were utilized to calculate the volumetric fraction of rubber in the swollen sample. The cross-link densities were assessed by Flory and Rehner equation [16]:

(2) $$\nu =\frac{-(\ln\left(1-V_{B}\right)+V_{B}+\chi \left(V_{B}{)}^{2}\right)}{\left(\rho_{B})(V_{0})(V_{B}^{\frac{1}{3}}-\frac{V_{B}}{2}\right)}\;$$

where *ν* is the cross-link density (mol cm^−3^), $\rho_{B}$ is the rubber density (g cm^−3^), and *V_B_* is the volume fraction of the rubber in swollen form. The values for the molar volume of toluene (*V*_0_) and the Flory–Huggins interaction parameter (*χ*) for natural/synthetic rubber and toluol were 106.3 cm^3^ mol^−1^ and 0.38, respectively.

The Mooney–Rivlin method was used to determine cross-link densities with data from tensile strength tests [17]. Equation (3) was used to obtain the linear regression graphs and network parameters [18].

(3) $$\sigma =\frac{F}{2A_{0}(\lambda -\lambda^{-2})}=C_{1}+\frac{1}{\lambda }C_{2}\;$$

where *F* is the force required in the vulcanized material; *A*_0_ is the cross-sectional area (mm^2^); λ is the extension rate (1 + ε), where ε is the strain; *C*_1_ the contribution of cross-linking units; *C*_2_ is the Mooney–Rivlin elastic constant, representing the contribution of fixed entanglements.

The material constant *C*_1_ can be used to calculate cross-link densities using Equation (4) [19]:

(4) $$ɳ=\frac{C_{1}}{RT}\;$$

where η is the cross-linking density (mol cm^−3^); R is the universal gas constant; and T is the absolute temperature (K).

### 2.6. Scanning Electron Microscopy (SEM)

The surface morphology of the fractured composites was examined using a Carl Zeiss EVO LS15 scanning electron microscope at an acceleration voltage of 20 kV. The specimens were coated with a thin layer of gold using a Quorum Q 150R ES sputter coater in Presidente Prudente, SP, Brazil.

### 2.7. Fourier Transform Infrared Spectroscopy in Attenuated Mode (FTIR-ATR)

FTIR-ATR spectroscopy was analyzed using a Bruker Vector 22 spectrometer in ATR (total attenuated reflection) mode localizated in Presidente Prudente, SP, Brazil. The analysis covered the wavelength range of 4000–400 cm^−1^ with a spectral resolution of 4 cm^−1^, and 32 scans were performed.

### 2.8. Tensile Strength Test

Tensile tests were performed on a universal testing machine at a rate of 500 mm min^−1^ with a load cell of 5 kN, according to ASTM D412-16 [20].

### 2.9. Shore A Hardness Test

The hardness of the composites was determined according to the ASTM D2240-15 standard, using an analog hardness meter on the Shore A scale, ranging from 0 to 100 [21].

### 2.10. Abrasion Loss

Abrasion loss was determined using Equation (5), according to ASTM D5963-22 [22], using equipment with an abrasion stroke equivalent to 40 m and a pressure on the sample in the cylinder of 5 N.

(5) $$PA=\frac{\Delta m\;S_{0}\;}{\rho \;S}\;$$

where *PA* is the abrasion loss (mm^3^/40 m); Δ*m* is the mass loss of the composite (mg); *S*_0_ is the theoretical attack index of sandpaper on standard rubber (200 ± 20 mg); *S* is the real attack index of the sandpaper on standard rubber (mg); and *ρ* is the density of the composite (mg mm^−3^).

### 2.11. Analysis of the Interactions between the Filler and the Polymer Matrix Using the Lorenz–Park Equation

The Lorenz–Park method was employed to ascertain the interaction between the polyurethane waste powder and the rubber mixture [23]. The parameters obtained from the swelling experiments were subsequently utilized in Equation (6) [24]:

(6) $$\frac{Q_{f}}{Q_{g}}={ae}^{-z}+b\;$$

where *Q* represents the amount of toluene absorbed per gram of rubber, and the subscripts *f* and *g* indicate the vulcanized composite with filler and gum, respectively. The variable *z* represents the ratio of the mass of the filler to the mass of the rubber, while *a* and *b* are constants. The value of *Q* was calculated using Equation (7):

(7) $$Q=\frac{w_{s}-w_{d}}{w_{r}\times 100/\;w_{F}}\;$$

where *w_s_* represents the weight of the composite when it reaches equilibrium, *w_d_* represents the weight of the composite when it is dry, *w_r_* represents the weight of the rubber in the dry composite, and *w_F_* represents the overall weight of the formulation.

## 3. Results and Discussion

### 3.1. Rheological Properties of Composites

Table 2 shows the values of the parameters measured in the composites during the rheometric tests: minimum torque (M_L_) and maximum torque (M_H_), torque variation (ΔM), pre-cure time (t_S1_), and optimal curing time (t_90_). Table 2 shows a slight increase in the minimum torque directly related to the viscosity of adding the filler. The increase in the values of the maximum torques is associated with the number of cross-links formed during the vulcanization process. The variation in torque also increases, which can be attributed to the formation of cross-links and their interactions with the filler.

The pre-curing time has gradually increased with the addition of the filler, which means that the rubber compound can be processed without premature vulcanization; and the optimal curing time remained practically stable with the incorporation of the filler, which may be related to the neutral nature of the polyurethane residue (pH 7–8) that does not influence the action of the accelerators [25].

### 3.2. Scanning Electron Microscopy (SEM) Analysis

**Figure 1:**
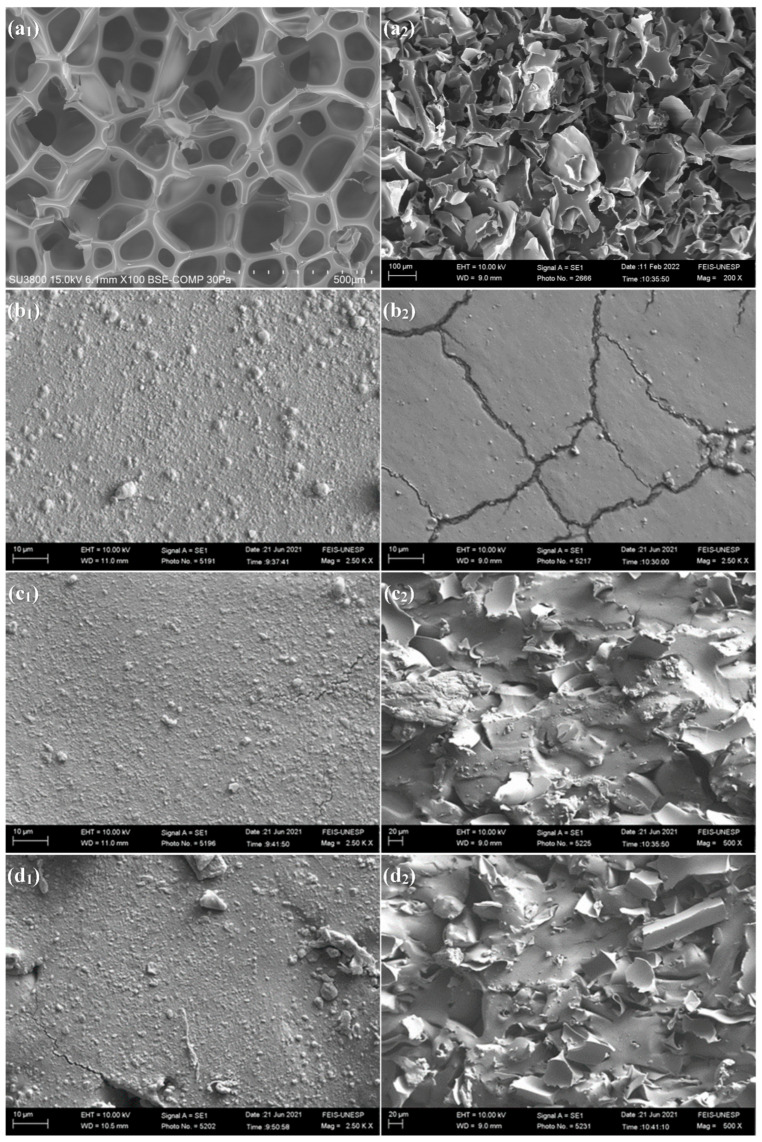

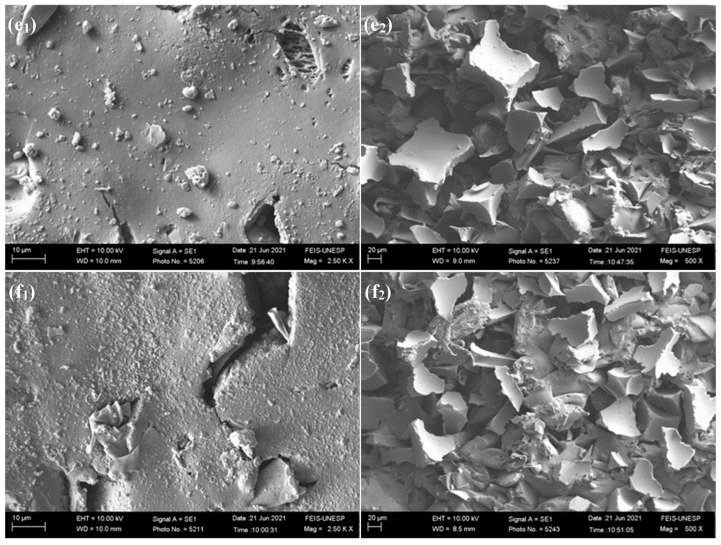
(**a_1_**) Polyurethane residue; (**a_2_**) micronized polyurethane; (**b_1_**) surface area of NSP 0 phr; (**b_2_**) fracture of NSP 0 phr; (**c_1_**) surface area of NSP 10 phr; (**c_2_**) fracture of NSP 10 phr; (**d_1_**) surface area of NSP 20 phr; (**d_2_**) fracture of NSP 20 phr; (**e_1_**) surface area of NSP 30 phr; (**e_2_**) fracture of NSP 30 phr; (**f_1_**) surface area of NSP 40 phr; and (**f_2_**) fracture of NSP 40 phr.

The scanning electron microscopy (SEM) images of polyurethane (PU) residues and rubber composites were obtained from test samples before and after mechanical processes such as micronization and fracture in tensile strength tests. These images are detailed in Figure 1a–f. In Figure 1(a_1_), it is possible to observe the laminar cut of a cellular structure belonging to the polyurethane foam. It is noted that the mechanical recycling process of rigid PU foam has a significant impact on its microstructure, highlighting sharp edges, as illustrated in Figure 1(a_2_).

The image in Figure 1(b_1_) clearly shows that a rougher texture characterizes the surface of the filler-free composite (0 phr). However, the homogeneity between the polymeric blends and vulcanizing agents is noteworthy. After the fracture process, as illustrated in Figure 1(b_2_), grooves resulting from plastic deformation become noticeable.

In the image of Figure 1(c_1_), associated with the composite containing 10 phr of polyurethane residue, it is possible to observe that the surface area resembles that of the unfilling compound. This observation arises from the uniformity of the mixture between the polymeric matrix and the filler. On the other hand, in the fracture zone, as evidenced in Figure 1(c_2_), strong adhesion of the polyurethane filler to the polymeric matrix is noticeable [26].

With 20 phr of polyurethane incorporated, the surface area depicted in Figure 1(d_1_) reveals scalings on the composite surface. This phenomenon may be associated with the onset of an excess of filler. In the rupture region, as illustrated in Figure 1(d_2_), it is still possible to observe a strong adhesion between the filler and the polymeric matrix.

By adding 30 and 40 phr of PU, represented in Figure 1(e_1_,f_1_), areas with deep depressions and that are exposed to the filler are noticeable. This phenomenon is associated with the excess filler present in the composition of the vulcanized material. The excessive increase in filler also tends to reduce the interaction between the filler and the polymeric matrix, initiating filler–filler interactions, which are weaker and result in a reduction in the material’s rupture resistance, as evidenced in the images of Figure 1(e_2_,f_2_).

### 3.3. Density, Hardness (Shore A), Abrasion Loss

The results of the tests for density, hardness on the Shore A scale, and abrasion loss are shown in Table 3. In general terms, the density of the material was consistent since the filler used has a low density, not significantly affecting the density of the composite.

In the results of the hardness tests shown in Table 3, it is observed that the increase in filler increased hardness due to the reinforcing nature of the added filler and the increase in the density of cross-links [27].

Table 3 also shows the results of the abrasion resistance test of the composites with and without polyurethane (PU) residue. Increased volume loss due to abrasion was observed with increasing filler, and the most significant volume loss occurred in the 40 phr composite. This phenomenon can be explained by the intense interaction between the filler particles in this composite, leading to agglomerates that generate surface stresses, increasing the stiffness of the composite material and reducing its elasticity [28]. On the other hand, considering the statistical error, it can be observed that the abrasion loss remained practically unchanged in the composites with 0, 10, and even 20 phr of polyurethane.

### 3.4. Tensile Strength Analysis

**Figure 2:**
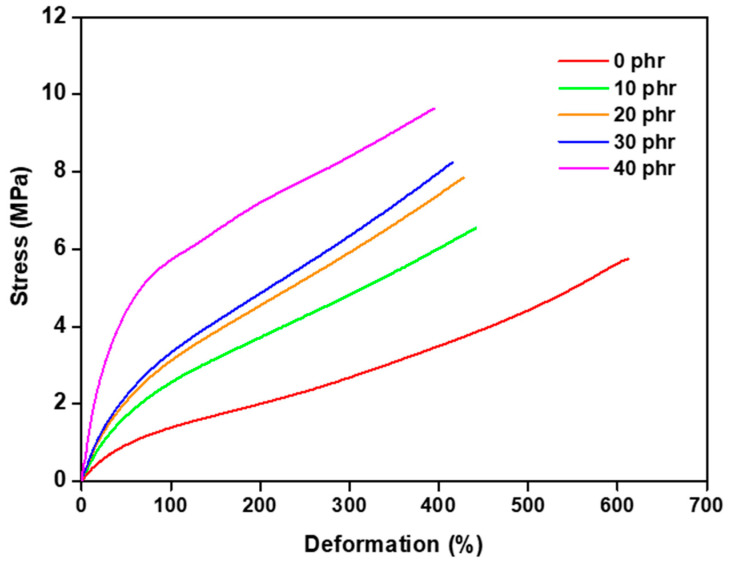
Tensile strength curves of NSP composites.

The profiles of the tensile strength curves are shown in Figure 2, and the values of the stress vs. strain results at break are shown in Table 4. A higher strain value and lower stress were observed in the reference sample, i.e., without filler. However, as the filler was increased, there was an increase in the maximum voltage values for the samples, and the optimum values were achieved in the samples with 30 phr. The increase in tension and the reduction in strain with the addition of filler can be attributed to the restriction of mobility in the rubber chains, stemming from the interfacial interaction between the filler and the matrix, as well as the stiffening of the composites [29].

In addition, the filler tends to form physical cross-linking points, which results in a higher tensile strength of the material. It can also be stated that the filler physically adheres to the polymer matrix, reinforcing it [30]. However, for the sample with 40 phr, a decrease in the strain was observed, indicating the saturation of the filler in the sample and, consequently, the loss of mechanical properties.

### 3.5. Analysis of Cross-Link Densities by the Organic Solvent Swelling Method—Flory–Rehner Method

The swelling tests in organic solvent allowed the quantification of cross-linking density in the samples, and these data are compiled in Table 5. The determination of cross-link densities through the swelling method in organic solvent until equilibrium, using the equation developed by Flory and Rehner, is an economical and common practice in the analysis of elastomeric materials such as rubber. When adding a filler, whether organic or inorganic, to the rubber formulation, it is observed that the amount of added filler is directly related to the increase in the cross-link density. This correlation can be explained by the following reasons:

Molecular interaction: The presence of fillers in the formulation may promote additional molecular interactions between filler particles and rubber matrix molecules. These interactions can favor the formation of more cross-links during the curing process.

Increased structural complexity: The introduction of fillers can increase the structural complexity of the rubber matrix, providing more sites for the formation of cross-links. This can occur due to the creation of additional reactivity sites in the presence of the filler.

### 3.6. Analysis of Cross-Link Densities Using Tensile Strength Results—Mooney–Rivlin Method

**Figure 3:**
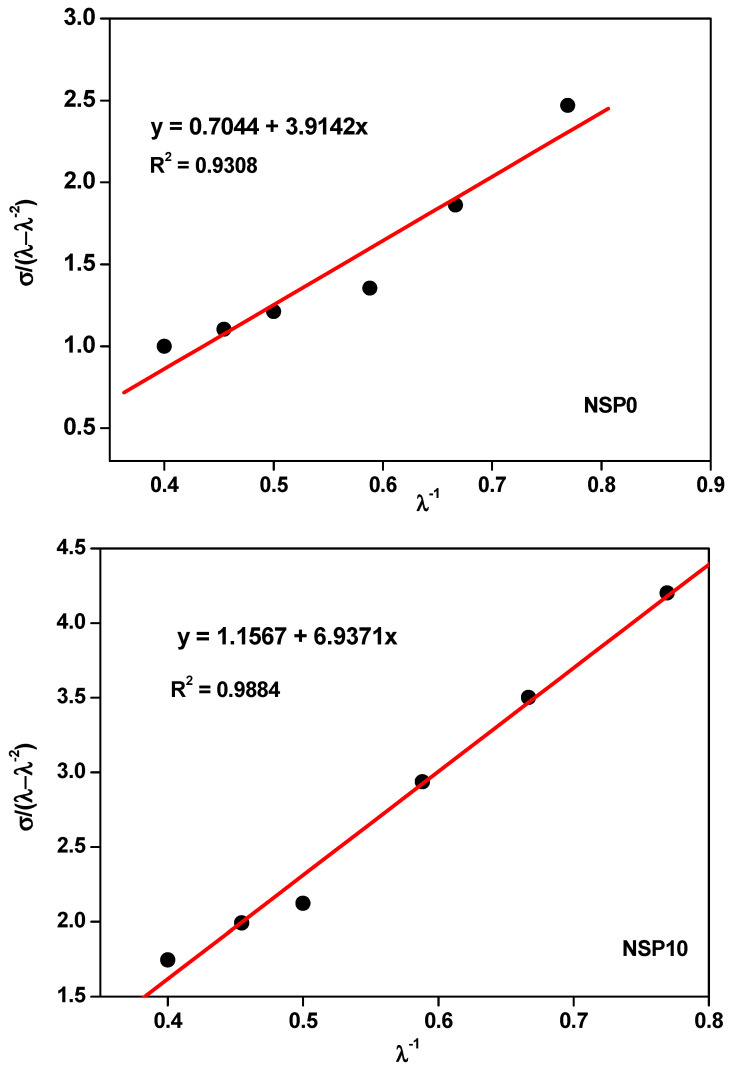

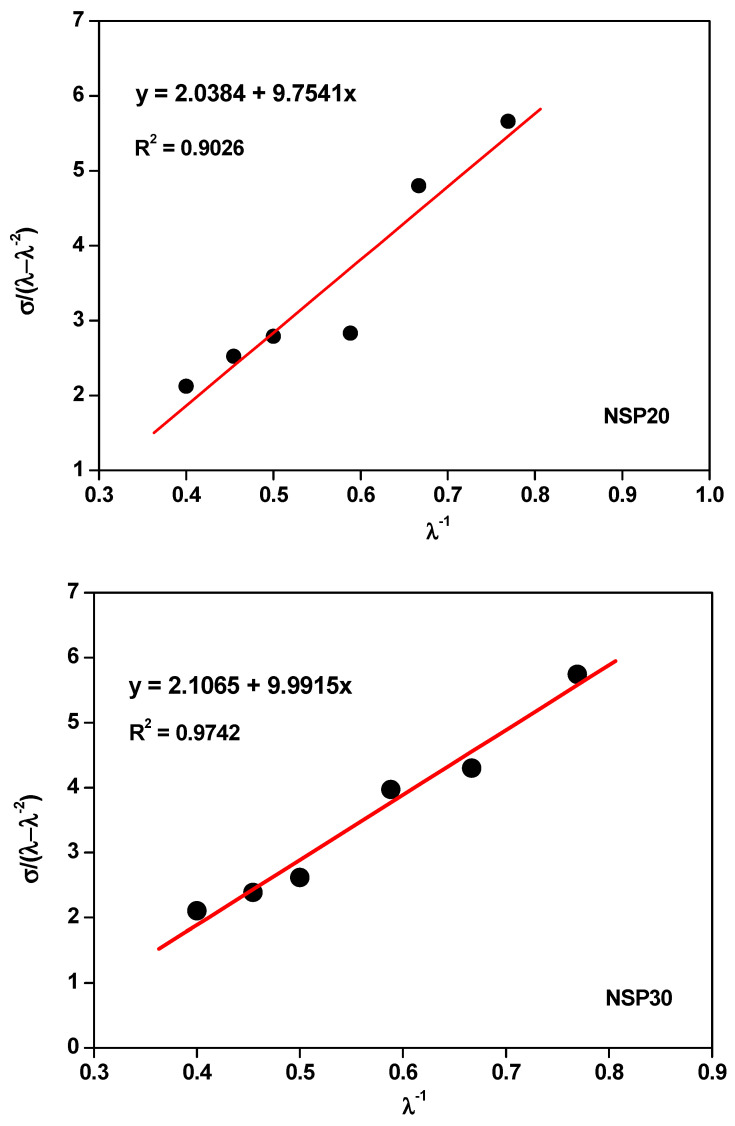

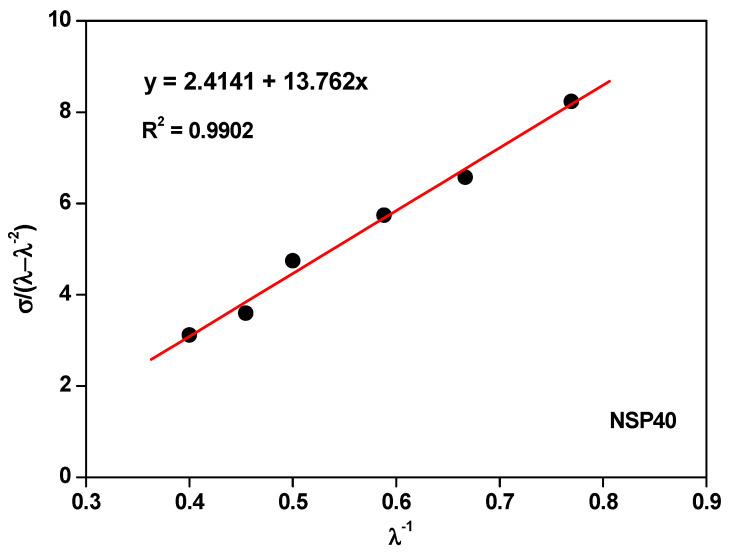
Plot of σ/(λ − λ^−2^) versus λ^−1^ of NSP composites.

Mechanical tensile strength tests determined cross-link densities. Points within the strain range between 30% and 150% (λ^−1^ ≈ 0.4–0.8) were selected. Linear regressions were performed to obtain the constants *C*_1_ and *C*_2_, as shown in Figure 3. These values were used to calculate each composite’s cross-links and specific constants, together with the cross-link densities presented in Table 6.

The constant *C*_1_, which represents the linear coefficient of the equation of the line, indicates the bonds formed in the composite and is directly related to the density of cross-links. It is observed that the increase in filler is associated with an increase in *C*_1_ and, consequently, with an increase in the density of cross-links. In addition, there is also an increase in the constant *C*_2_, which represents the slope coefficient and is related to the intermolecular forces in the polymer chain. These results show the intense interaction between the filler and the matrix, indicating the reinforcement provided by the filler and the cross-links.

### 3.7. Analysis of Interfacial Interaction Using the Lorenz–Park Methodology

**Figure 4:**
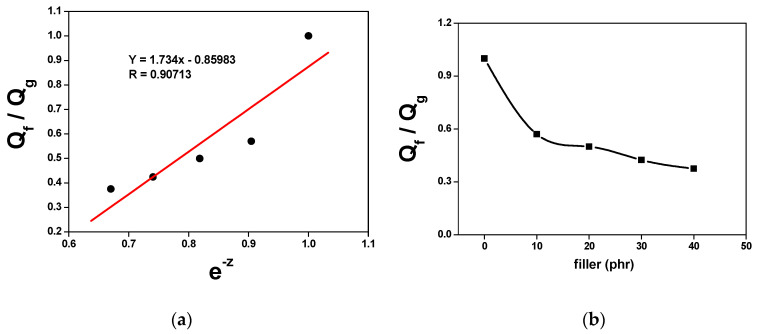
(**a**) Variation in *Q_f_*/*Q_g_* versus *e*^−*z*^ in the composites and (**b**) filler effect on *Q_f_*/*Q_g_* in the NSP composites.

The *Q_f_*/*Q_g_* coefficients are derived using the suggested methodological approach and are plotted in Figure 4. The curve *Q_f_*/*Q_g_* by *e*^(−*z*)^ delineates the linear relationship with a positive slope. The constant parameters *a* and *b*, with specific numerical values of 1.734 and 0.85983, respectively, are identified, along with a correlation coefficient (R) equivalent to 0.91. These values show the intense interaction between the filler and the matrix, as highlighted by Abhisha et al. [31]. A constant ‘*a*’ greater than 0.7 indicates a substantial interaction between the polyurethane and the rubber, inhibiting solvent penetration into the composite.

In addition, considering that all composites are manufactured from the same elastomer, using an identical cross-linking system, it is valid to state that the observed reduction in the *Q_f_*/*Q_g_* ratio, as illustrated in Figure 4, due to the increase in the proportion of polyurethane, confirms a significant improvement in the interaction between the filler and the rubber. Furthermore, this is reflected in the mechanical properties of the composites. Higher values for the *Q_f_*/*Q_g_* ratio indicate more subtle interactions between the filler and the rubber, as highlighted by Hayeemasae, Nabil, and Ismail [32].

### 3.8. Spectra Analysis in the Infrared with Fourier Transform in Attenuated Reflectance Mode

**Figure 5:**
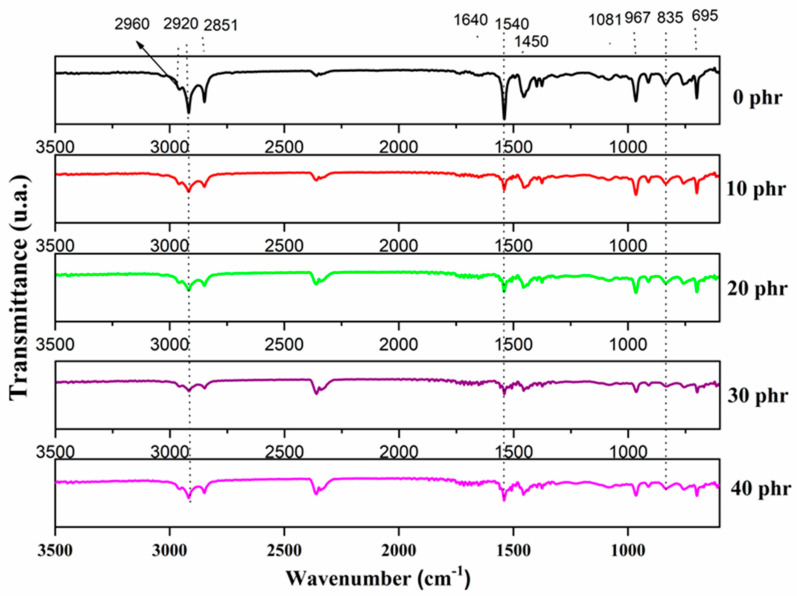
FTIR spectra of NSP composites.

Figure 5 shows the infrared spectra of the pure gum (0 phr) NSP composites and the filler composites. All samples generally had the same bands but with different intensities. For the band observed around 697 cm^−1^, aromatic substitution (δ_C-H_) is attributed. In addition, the band around 835 cm^−1^ is related to the bending vibrations of the CH group. Bands in the 950–1380 cm^−1^ range are associated with the functional groups present in rubber [33]. Between 950 and 1150 cm^−1^, the rocking vibrations of the CH_3_ group occur, while the vibrational modes of twisting and shaking occur between 1150 and 1380 cm^−1^ [34].

Interestingly, for pure gum (0 phr), there is a low-intensity band around 1080 cm^−1^, and as the filler increases, this intensity decreases. This band is related to the symmetrical elongation vibrations of the C-S-C groups, which are the cross-links. As the filler increases, the absorption rate changes for these composites. Thus, the material has a better absorption in the sample without reinforcing the filler. The band noted at around 1452 cm^−1^ is attributed to the axial deformation modes of CH_3_ [35]. The band around 1540 cm^−1^ refers to the elongation oscillation of the conjugate double bond attached to the methyl group. The slight variation around 1650 cm^−1^ represents the C=C band referring to isoprene and butadiene [36].

### 3.9. Application of Composite

**Figure 6:**
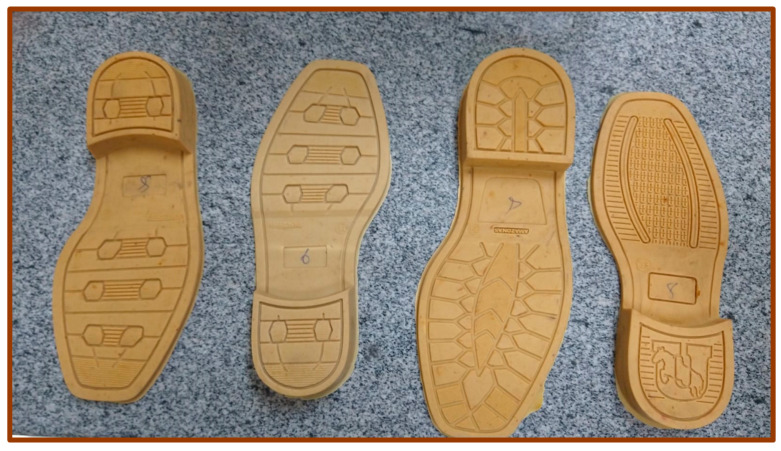
Footwear sole made from natural and synthetic rubbers with polyurethane residue.

Figure 6 shows the molded footwear sole at Grupo Arizona Componentes para Calçados LTDA, which incorporated the samples into the production line. The new compound for shoe soles presented appropriate lightness and hardness, suitable for casual shoes.

## 4. Conclusions

Based on the results obtained in this study, it was possible to analyze the influence of micronized polyurethane on the NR/SBR polymeric matrix. The investigation of the interaction between the filler and the matrix confirmed the reinforcing effect of polyurethane on the polymer blend, increasing the mechanical properties as the filler increased.

The quantification of the cross-link density employing the swelling assay indicated an increase proportional to the filler due to the ability of the filler to act as a barrier against toluene. In the tensile strength test, a variation in the behavior of the composite was observed with the addition of the filler. The increase in the filler resulted in higher stress, evidencing the reinforcing characteristic of the filler in the matrix. In addition, the composites met the minimum requirements for industrial applications. However, with the addition of 40 phr in the polymer matrix, there was a reduction in the deformation, indicating the saturation of the material and the beginning of the loss of physical properties.

Fourier transform infrared spectroscopy (FTIR) analysis allowed the identification of the characteristic bands of natural rubber and SBR in the composition. No new bands were observed, indicating the absence of a chemical reaction between the polymeric blend and the polyurethane residue. The materials generally showed good homogeneity since all samples showed the same bands, varying only in intensity.

Scanning electron microscopy (SEM) revealed the surface of the composites and fracture regions. With the increase in the filler, there was an increase in the presence of particles in the images, which was more evident in the 30 and 40 phr samples, where irregularities were observed in the composite. Furthermore, this can be attributed to the strong interaction between the filler and the matrix and the saturation of the filler in the matrix, respectively.

The 10 phr composite showed the lowest volume loss in the abrasion resistance test due to the excellent filler dispersion. In addition, the hardness achieved by the composite met the requirements for sole applications, as it establishes a minimum hardness of 55, which can reach 75–80 on the Shore A scale, depending on the specific application.

After analyzing and correlating the results obtained, it was possible to verify that, for this specific formulation, the composite containing 30 phr of polyurethane presented the best performance. This composite showed consistent results, with no filler saturation in the polymeric matrix. In addition, it is possible to conclude that the composites satisfactorily met the requirements established by the footwear industry.

Thus, the potential use of this elastomeric blend, which incorporates polyurethane as a reinforcing filler, is confirmed. It is worth mentioning that the polyurethane used comes from the environmental sector of the refrigeration industry, thus evidencing the feasibility of its application in footwear, contributing to more sustainable practices in the industry.

## Figures and Tables

**Table 1:** Formulation of NR-SBR compounds with different proportions of PU residue.

Materials	Quantity of Components in phr				
	NSP_0_ *	NSP_10_ *	NSP_20_ *	NSP_30_ *	NSP_40_ *
NR	50	50	50	50	50
SBR 1502	50	50	50	50	50
zinc oxide	4	4	4	4	4
Stearic Acid	2	2	2	2	2
Polyurethane residue	0	10	20	30	40
sulphur	2.5	2.5	2.5	2.5	2.5
MBTS accelerator	1	1	1	1	1
TMTD Accelerator	1	1	1	1	1

* NSP (NR, SBR, PU).

**Table 2:** Rheometric parameters of NSP composites.

NSP Composites	M_L_ (dNm)	M_H_ (dNm)	ΔH = M_H_ − M_L_ (dNm)	t_S1_ (min)	t_90_ (min)
0 phr	0.44 ± 0.01	3.41 ± 0.32	2.97 ± 0.41	1.48 ± 0.21	3.25 ± 0.18
10 phr	0.46 ± 0.01	4.17 ± 0.21	3.71 ± 0.24	1.63 ± 0.14	2.68 ± 0.15
20 phr	0.61 ± 0.02	4.99 ± 0.3	4.38 ± 0.33	1.67 ± 0.11	2.77 ± 0.21
30 phr	0.59 ± 0.01	5.73 ± 0.52	5.14 ± 0.51	1.78 ± 0.2	2.9 ± 0.17
40 phr	0.63 ± 0.01	5.91 ± 0.43	5.28 ± 0.5	2.05 ± 0.31	3.42 ± 0.5

**Table 3:** Density, hardness (Shore A), and abrasion loss of NSP composites.

NSP Composites	Density (g cm^−3^)	Hardness (Shore A)	Abrasion Loss (mm^3^/40 m)
0 phr	1.005	50 ± 3	282 ± 13
10 phr	1.005	55 ± 2	295 ± 18
20 phr	1.007	64 ± 3	307 ± 22
30 phr	1.027	70 ± 2	400 ± 31
40 phr	1.040	75 ± 4	431 ± 24

**Table 4:** Table of stress vs. strain results values at break of NSP composites.

NSP Composites	Stress (MPa)	Strain (%)
0 phr	5.81 ± 0.31	615 ± 21
10 phr	6.54 ± 0.43	442 ± 18
20 phr	7.92 ± 0.27	428 ± 31
30 phr	8.23 ± 0.44	417 ± 19
40 phr	9.66 ± 0.25	395 ± 25

**Table 5:** Density and number of cross-links of NSP—Flory–Rehner composites.

NSP Composites	Cross-Link Density 10^−4^ (mol cm^−3^)	Number of Cross-Links 10^20^ (cm^3^)
0 phr	1.90	1.1457
10 phr	1.95	1.1458
20 phr	2.04	1.2273
30 phr	2.43	1.4606
40 phr	2.65	1.6147

**Table 6:** Density and numbers of cross-links of NSP—Mooney–Rivlin composites.

NSP Composites	Cross-Link Density 10^−4^ (mol cm^−3^)	Number of Cross-Links 10^20^ (cm^3^)	*C*_1_	*C*_2_
0 phr	3.08	1.86	0.7044	3.9142
10 phr	4.67	2.82	1.1567	6.9371
20 phr	8.23	4.96	2.0384	9.7541
30 phr	8.50	5.13	2.1065	9.9915
40 phr	9.74	5.88	2.4141	13.7620

## Data Availability

The data are not publicly available due to privacy.
